# *Navigare necesse est*: The transorbital route to intraparenchymal temporal lesions

**DOI:** 10.1007/s10143-026-04402-x

**Published:** 2026-08-25

**Authors:** Edoardo Agosti, Lorenzo Gelmini, Francesco Corrivetti, Giorgio Iaconetta, Pascal Lavergne, Marco Maria Fontanella, Pierre-Olivier Champagne, Doo-Sik Kong, Matteo De Notaris

**Affiliations:** 1 Division of Neurosurgery, Department of Medical and Surgical Specialties, Radiological Sciences and Public Health, University of Brescia, Piazza Spedali Civili 1, Brescia, 25123 Italy; 2 Laboratory of Neuroanatomy, EBRIS Foundation, Salerno, Italy; 3 Unit of Neurosurgery, University Hospital San Giovanni di Dio e Ruggi d’Aragona, University of Salerno, Salerno, Italy; 4 Division of Neurosurgery, Department of Surgery, Sacré-Coeur Hospital, Université de Montréal, Montréal, QC Canada; 5 Departement of Neurosurgery, CHU de Québec - Université Laval, Québec, QC Canada; 6 Department of Neurosurgery, Samsung Medical Center, Sungkyunkwan University School of Medicine, Seoul, Republic of Korea

**Keywords:** Transorbital approach, Temporal lobe, Mediobasal temporal region, Systematic review

## Abstract

During the last years, the endoscopic transorbital approaches (ETOAs) have emerged as a minimally invasive alternative to traditional microsurgical transcranial approaches (MTAs) for accessing selected intraparenchymal temporal lesions, particularly those located in the temporopolar (pTL) and mediobasal temporal regions (mbTR). While certain MTAs approaches with a lateral-to-medial trajectory toward the mesial temporal lobe may pose a higher risk of optic radiation injury and consequent visual field (VF) deficits, ETOAs offer an anterior-to-posterior trajectory that may minimize such risks. This systematic review aims to assess the safety, efficacy, and technical aspects of ETOAs highlighting also the preservation of temporalis muscle integrity as an additional advantage of the transorbital approach. A systematic literature search was conducted according to PRISMA guidelines using PubMed, Scopus and Ovid MEDLINE to identify clinical studies evaluating ETOA for intraparenchymal temporal lesions. Inclusion criteria encompassed studies reporting clinical and/or surgical outcomes, including extent of resection (EoR), postoperative complications, and VF deficits. Descriptive statistics were applied. Additionally, illustrative clinical cases were reviewed to highlight technical nuances. Thirteen studies met inclusion criteria, encompassing 41 patients. Most lesions were in the mbTR (87.8%), with a smaller number involving the temporal pole (12.2%). Gross total resection was achieved in 27 patients (73.0%), subtotal resection in 5 (13.5%), partial resection in 3 (8.1%), and biopsy alone in 2 (5.4%). Postoperative visual outcomes were favorable: of 29 patients with available data, 23 (79.3%) preserved their preoperative intact VF and none experienced postoperative VF deterioration. Complications were rare, with a single case (2.6%) of CSF leak and no instances of permanent cranial nerve deficits. Endoscopic transorbital route is a promising and versatile approach for selected intraparenchymal temporal lesions, offering a function-preserving alternative to conventional MTAs. Its anterior-to-posterior surgical corridor along the temporo-insular axis may facilitate reduced subcortical disruption, potentially allowing optic radiation sparing while preserving temporalis muscle integrity, further support its integration into neurosurgical practice. Clinical trial number: not applicable

**Supplementary Information:**

The online version contains supplementary material available at 10.1007/s10143-026-04402-x.

## Introduction

In the evolving landscape of skull base techniques, minimally invasive endoscopic approaches have expanded the boundaries of neurosurgical access to deep-seated brain structures [1–6]. Among these, the endoscopic transorbital approaches (ETOAs) have emerged as a particularly promising route [1, 7–9]. Originally developed for paramedian extra-axial skull base lesions - including tumors of the lateral cavernous sinus, Meckel’s cave, petrous apex, and anterior cranial base - ETOAs have since evolved to encompass select intraparenchymal temporal lesions [10–21].

The earliest applications of ETOA beyond the skull base targeted the temporal pole, where a straight anterior trajectory allowed resection of tumors such as gliomas and cavernous malformations, with minimal disruption to lateral neocortex [22, 23]. Encouraged by these results, surgeons progressively advanced the technique posteriorly, toward the mediobasal temporal region (mbTR), an anatomically intricate zone historically addressed through more invasive transcranial approaches [24–27]. More recently, interest has extended even further, with a preliminary case exploring ETOA access to the insular region, marking a significant evolution in the corridor’s potential [28].

As ETOA has expanded from skull base to intraparenchymal targets along the temporo-insular axis, favorable CSF leak rates have been reported for anterior and middle skull base lesions requiring dural opening, reinforcing the technique’s versatility and its growing role in contemporary neurosurgery [29].

This systematic review aims to comprehensively examine the emerging role of ETOAs in the management of intraparenchymal temporal lesions, focusing on extent of resection (EoR), visual preservation and complication rates. Furthermore, by presenting illustrative cases, we aim to contextualize the technical nuances, anatomical challenges, and real-world applicability of this approach [Illustrative cases 1 and 2]. A particular emphasis is also put on the evolution from skull base to parenchymal targets, the transition from temporopolar to mediobasal involvement and the integration of neuroimaging adjuncts for safe surgical planning.

## Materials and methods

### Literature review

This systematic review was conducted in accordance with the Preferred Reporting Items for Systematic Reviews and Meta-Analyses (PRISMA) guidelines. Two reviewers (E.A. and L.G.) independently performed a comprehensive search of the PubMed, Scopus and Ovid MEDLINE databases. The initial search was conducted on March 2, 2025, with a final update on April 18, 2025.

The search strategy utilized combinations of keywords and MeSH terms, including “endoscopic transorbital approach,” “ETOA,” “temporal lobe,” “intraparenchymal lesions,” “mediobasal temporal region,” “temporal pole,” “insular cortex,” “tumor resection,” and “neurosurgery.” Boolean operators (AND/OR) were used to structure the query as follows: (“endoscopic transorbital approach” OR “ETOA”) AND (“temporal lobe” OR “mediobasal temporal” OR “temporal pole” OR “insula”) AND (“tumor” OR “resection” OR “surgery”) (Supplementary Material 1). Additional relevant studies were identified through manual screening of reference lists from selected articles.

Studies were eligible for inclusion if they were published in English, included clinical data, and focused on the use of ETOA for the surgical treatment of intraparenchymal temporal lesions. Only studies reporting clinical or surgical outcomes, such as extent of resection (EoR), visual function, or complication rates, were considered. Articles were excluded if they exclusively addressed skull base lesions without parenchymal extension, non-lesional epilepsy, or if they were review articles, editorials, cadaveric studies, conference abstracts, or non-English publications. Studies lacking clearly reported outcomes or methodology were also excluded.

All references were managed using EndNote X9, and duplicates were removed. Titles and abstracts were screened independently by two reviewers (E.A. and L.G.), and any discrepancies were resolved by discussion with a third reviewer (M.D.N.). Full-text screening was subsequently performed on all studies that met the initial eligibility criteria.

### Data extraction

Data were systematically extracted from each included study using a standardized template. Extracted variables included authorship, year of publication, study design, number of patients, lesion type and anatomical location (temporal pole or mediobasal temporal region), clinical presentation, surgical approach and extent of resection, as well as pre- and postoperative visual outcomes. Additional data were collected on postoperative complications, such as cerebrospinal fluid (CSF) leaks, permanent cranial nerves deficits, cosmetic issues, and on the use of any intraoperative adjuncts, including neuronavigation or diffusion tensor imaging (DTI) tractography.

### Outcomes

The aim of this systematic review was to characterize the overall role and technical feasibility of ETOA in the surgical management of intraparenchymal temporal lesions.

The primary outcome was the EoR rate. Secondary outcomes included the incidence of postoperative visual function preservation and the rate of surgery-related complications. We defined gross-total resection (GTR) as no evidence of residual tumor on the postoperative magnetic resonance imaging (MRI), subtotal resection (STR) as more than or equal to 90% of tumor removal on the postoperative MRI, and partial resection (PR) as less than 90% of tumor removal on the postoperative MRI. Postoperative visual field (VF) was defined as follows: “preserved” if the preoperative VF was intact and no new deficits were reported after surgery; “stable” if a preexisting deficit showed no further postoperative deterioration, or if the preoperative VF was unavailable and no new deficits emerged after surgery; and “worsened” if new VF deficits were reported or existing ones progressed, regardless of the preoperative VF status.

Two illustrative cases were also reviewed to highlight anatomical variations and technical nuances in the application of ETOAs to different lesion locations.

### Risk of bias assessment

The Newcastle-Ottawa Scale (NOS), which rates studies according to selection, comparability, and outcome assessment categories, was used to evaluate the quality of the included observational studies (Supplementary Material 2). Every study received a score of up to nine points. Studies that received less than seven points were deemed to be of low quality and were not included in the analysis. To interpret any bias resulting from research quality, the remaining papers were taken into consideration for subgroup analyses and narrative synthesis, irrespective of their score. Each study’s specific NOS scores are listed in Supplementary Material 3.

### Statistical analysis

Descriptive statistics were used to summarize patient demographics, lesion characteristics, surgical outcomes, and complication rates. Data were reported using ranges, medians, and proportions. All statistical analyses were performed using R statistical software (version 4.2.0) (https://www.r-project.org).

## Results

###  Literature review

**Fig. 1:**
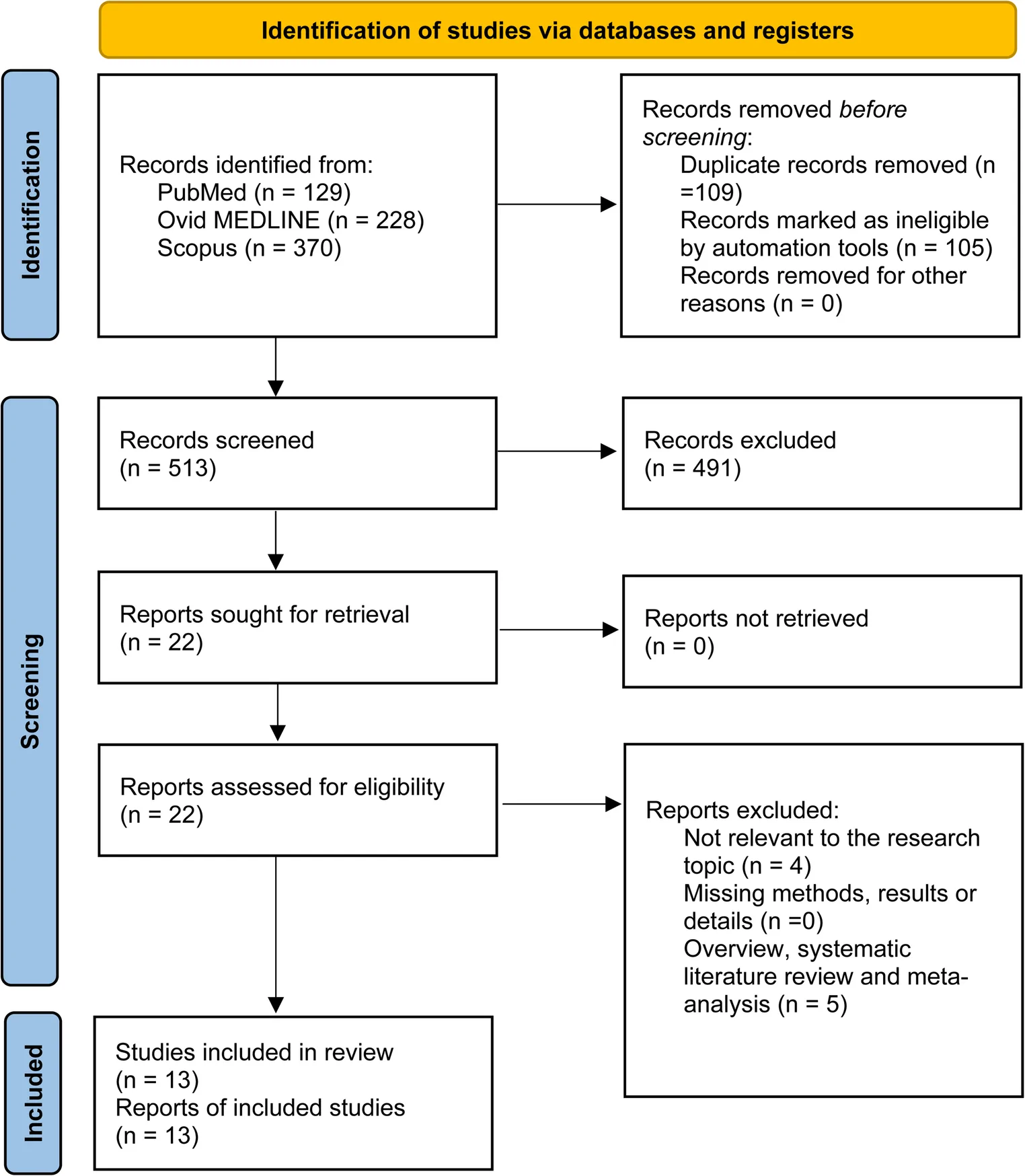
Flowchart according to the PRISMA statement

A total of 513 papers were identified after duplicate removal. After title and abstract analysis, 22 articles were identified for full-text analysis. Eligibility was assessed for 22 articles and ascertained for 13 articles [22–27, 30–36]. The remaining 9 articles were excluded because not relevant to the research topic (*n* = 4) or systematic literature review or meta-analysis (*n* = 5) [28, 29, 37–43] (Fig. 1) shows the flowchart according to the PRISMA statement.

This systematic review included a total of 41 patients who underwent ETOAs for intraparenchymal temporal lesions.

The median age at intervention was 48 years (35.5–62.5; *n* = 31).Among patients with available data, 13 of 38 were female (34.2%).

Regarding pathological diagnoses, the most frequent were low-grade gliomas (LGG) in 9 patients (21.9%), followed by glioblastomas (GBM) in 7 patients (17.1%).

Anaplastic astrocytomas (AA) and Cerebral cavernous malformations (CCM) were identified in 6 patients each (14.6%), rarer pathologies included gliosis (*n* = 2, 4.9%) and metastases from renal cell carcinoma (MTX RCC, *n* = 2, 4.9%). Lastly, various singular lesions such as adenoid cystic carcinoma (ACC), anaplastic oligodendroglioma (AO), dermoid cyst (DC), hemangiopericytoma (HPC), metastases from pharyngeal carcinoma (MTX PC), as well as multinodular and vacuolating neuronal tumor (MVNT), pleomorphic xanthoastrocytoma (PXA), plasma cell neoplasm (PCN), and teratoma, were observed; each accounting for 2.4% (*n* = 1) of cases.

Lesion location was predominantly in the mediobasal temporal region (mbTR) in 36 patients (87.8%), while the temporal pole (pTL) was affected in 5 cases (12.2%).

Clinical presentation prior to surgery was available for 28 patients regarding VF status, with 25 (89.3%) exhibiting intact preoperative VF and 3 (10.7%) presenting worsened VF.

Among 31 patients assessed, 19 (61.3%) experienced seizures. Other observed symptoms included headache (*n* = 6, 20%), cognitive impairment (*n* = 3, 10%), aphasia (*n* = 2, 6.7%) and facial numbness (*n* = 1, 3.3%), considering the group of 30 patients with available clinical data (Tables 1 and 2).

**Table 1:** Anamnestic, pathological, and clinical data of studies included in the systematic review

Author and year	Baseline data		Anamnestic data *N*				Pathology *N* (%)		Clinical Presentation *N* (%)			
	Country	Study period	Patient Cohort	Included patients	Mean Age at Intervention (range)	Female (%)	Diagnosys	Location	Preop VF		Seizures	Other sympthoms
Chen et al., 2015 [27]	United States	NA	2	2	51.5 (43–60)	1 (50)	Gliosis 2 (100)	mbTR 2 (100)	Intact 2 (100)		2 (100)	0 (0)
Jeon et al., 2018 [30]	Republic of Korea	2016–2018	9	1	54	0 (0)	MTX RCC 1 (100)	mbTR 1 (100)	NA		NA	NA
Lee et al., 2019 [31]	Republic of Korea	2015–2018	21	1	60	1 (100)	DC 1 (100)	mbTR 1 (100)	Intact 1 (100)		0 (0)	0 (0)
Gerges et al., 2020 [23]	United States, Egypt	NA	1	1	77	1 (100)	GBM 1 (100)	pTL 1 (100)	NA		NA	NA
Yoo et al., 2021 [22]	Republic of Korea, Republic of Singapore	2017–2019	22	7	NA	2 (28.6)	AA 3 (42.9) GBM 1 (14.3) LGG 1 (14.3) PCN 1 (14.3) HPC 1 (14.3)	mbTR 6 (85.7) pTL 1 (14.3)	NA		NA	NA
Park et al., 2021 [24]	Republic of Korea	2018–2020	7	7	56.7 (23–74)	0 (0)	AA 3 (42.9) GBM 1 (14.3) LGG 3 (42.9)	mbTR 7 (100)	Intact 7 (100)		4 (57.1)	Headache 2 (28.6) Cognitive impairment 1 (14.3)
Lee et al., 2022 [32]	Republic of Korea	2015–2019	19	1	20	0 (0)	Teratoma 1 (100)	mbTR 1 (100)	NA		NA	NA
Tanoue et al., 2024 [33]	Japan	2016–2023	1	1	43	0 (0)	CCM 1 (100)	pTL 1 (100)	Intact 1 (100)		1 (100)	0 (0)
Carbone et al. 2024 [34]	Italy, Germany	2022	20	1	48	0 (0)	MTX PC 1 (100)	pTL 1 (100)	NA1		0 (0)	0 (0)
Iwami et al., 2024 [25]	Japan	NA	2	2	NA	NA	GBM 2 (100)	mbTR 2 (100)	Intact 2 (100)		0 (0)	Cogntive impairment 2 (100) Aphasia 2 (100)
Torregrossa et al., 2025 [35]	United States, Italy	2015–2023	40	1	NA	NA	CCM 1 (100)	mbTR 1 (100)	NA		1 (100)	NA
Jeon et al., 2025 [26]	Republic of Korea	2017–2022	15	15	43 (22–76)	7 (46.7)	LGG 5 (33.3) CCM 3 (20) GBM 2 (13.3) MVNT 1 (6.7) PXA 1 (6.7) AO 1 (6.7) ACC 1 (6.7) MTX RCC 1 (6.7)	mbTR 15 (100)	Intact 12 (80) Worsened 3 (20)		10 (66.7)	Headache 4 (26.7) Facial numbness 1 (6.7)
Maghalashvili et al., 2025 [36]	Italy, Georgia	NA	1	1	31	1 (100)	CCM 1 (100)	pTL 1 (100)	NA		1 (100)	0 (0)

**Table 2:** Summary of main anamnestic, pathological, and clinical data

TOTAL PATIENTS *N*			41				
ANAMNESTIC DATA N	Median Age at intervention (IQR)				48 (35.5–62.5)		
			**TOT**		**31**		
	Female (%)		13 (34.2)				
			**TOT**		**38**		
PATHOLOGY N (%)	Diagnosys	LGG			9 (21.9)		
		GBM			7 (17.1)		
		AA			6 (14.6)		
		CCM			6 (14.6)		
		Gliosis			2 (4.9)		
		MTX RCC			2 (4.9)		
		ACC			1 (2.4)		
		AO			1 (2.4)		
		DC			1 (2.4)		
		HPC			1 (2.4)		
		MTX PC			1 (2.4)		
		MVNT			1 (2.4)		
		PXA			1 (2.4)		
		PCN			1 (2.4)		
		Teratoma			1 (2.4)		
		**TOT**			**41**		
	Location	mbTR	36 (87.8)				
		pTL	5 (12.2)				
		**TOT**			**41**		
CLINICAL PRESENTATION N (%)	Preop VF	Intact	25 (89.3)				
		Worsened	3 (10.7)				
		**TOT**			**28**		
	Seizures	19 (61.3)					
		**TOT**			**31**		
	Headache	6 (20.0)					
	Cognitive impairment	3 (10.0)					
	Aphasia	2 (6.7)					
	Facial numbness	1 (3.3)					
		**TOT**			**30**		

Regarding surgical approach, the superior eyelid transorbital endoscopic approach (SETOA) alone was used in 20 patients (48.8%), and a combined SETOA with lateral orbitotomy (SETOA + LO) was adopted in the same number of patients (*n* = 20, 48.8%). Lastly, the inferolateral transorbital endoscopic approach (ILTEA) was applied in a single patient (2.4%). Notably, in all these approaches, temporalis muscle integrity was preserved.

Among the 37 patients for whom EoR data were available, GTR was achieved in 27 patients (73.0%), STR in 5 patients (13.5%), PR in 3 patients (8.1%), and biopsy alone was performed in 2 cases (5.4%).

Postoperative complications were infrequent: a CSF leak occurred in 1 case (2.6%), while no permanent cranial nerve deficits or diplopia were reported (0%, *n* = 35). Other complications included one case each (2.6%) of postoperative stroke, severe bleeding, pseudomeningocele, and hematoma. Regarding functional outcomes, postoperative VF assessment was available in 29 patients, of whom 23 (79.3%) preserved their preoperative intact VF, 6 (20.7%) remained stable, and none experienced worsened VF (0%).

Among the 29 patients with available postoperative VF outcomes, 15 (51.7%) underwent VF assessment using both clinical ophthalmological examination and OR tractography to confirm intact OR. Two patients (6.9%) were assessed only clinically, and VF assessment was unavailable for the remaining 12 patients.

Seizure control, as measured by the Engel Outcome Scale, was achieved in all evaluable patients (*n* = 4) with classification as Engel Class I. No cosmetic issues were reported in the included studies. However, only six studies explicitly reported patients’ aesthetic outcomes in the results section, whereas most studies provided postoperative photographs documenting the surgical scar.

The mean follow-up duration across studies was 14.2 months, ranging from 2 to 35 months (Tables 3 and 4).

**Table 3:** Surgical and clinical outcomes data of studies included in the systematic review

Author and year	Surgical ApproachN (%)	Outcome *N* (%)						Mean FU in months (range)
		Surgical				Clinical		
		EoR	CSF leak	Permanent CNs deficit (III, IV, VI) and/or diplopia	Other complications	Postop VF	Seizure Control -Engel Outcome Scale	
Chen et al., 2015 [27]	SETOA 1 (50) SETOA + LO 1 (50)	STR 1 (50) PR 1 (50)	0 (0)	0 (0)	Pseudomeningocele 1 (50)	Preserved 2 (100)	2 (100) - Class I	12
Jeon et al., 2018 [30]	SETOA 1 (100)	GTR 1 (100)	0 (0)	0 (0)	0 (0)	Stable 1 (100)	0 (0)	NA
Lee et al., 2019 [31]	SETOA 1 (100)	GTR 1 (100)	0 (0)	0 (0)	NA	Preserved 1 (100)	0 (0)	9
Gerges et al., 2020 [23]	ILTEA 1 (100)	Biopsy 1 (100)	NA	0 (0)	NA	Stable 1 (100)	NA	NA
Yoo et al., 2021 [22]	SETOA 7 (100)	GTR 3 (50) STR 3 (50) NA 1	1 (6.7)	NA	Stroke 1 (14.3) Severe bleeding 1 (14.3)	NA	NA	NA
Park et al., 2021 [24]	SETOA 7 (100)	GTR 6 (85.7) STR 1 (14.3)	0 (0)	0 (0)	Postoperative hematoma of the surgical bed (resolved) 1(14.3)	Preserved 7 (100)	NA	22.9 (11- 29.7)
Lee et al., 2022 [32]	SETOA + LO 1 (100)	GTR 1 (100)	0 (0)	0 (0)	0 (0)	NA	NA	NA
Tanoue et al., 2024 [33]	SETOA + LO 1(100)	GTR 1(100)	0 (0)	0 (0)	0 (0)	Preserved 1 (100)	1 (100) - Class I	16
Carbone et al. 2024 [34]	SETOA 1(100)	Biopsy 1(100)	0 (0)	0 (0)	0 (0)	NA	0	NA
Iwami et al., 2024 [25]	SETOA + LO 2 (100)	NA	0 (0)	0 (0)	0 (0)	NA	0 (0)	10 (2–18)
Torregrossa et al., 2025 [35]	SETOA 1 (100)	NA	NA	0 (0)	NA	NA	NA	NA
Jeon et al., 2025 [26]	SETOA + LO 15 (100)	GTR 13 (86.7) PR 2 (13.3)	0 (0)	0 (0)	0 (0)	Preserved 12 (80) Stable 3 (20)	NA	12 (6–35)
Maghalashvili et al., 2025 [36]	SETOA 1 (100)	GTR 1 (100)	0 (0)	0 (0)	0 (0)	Stable 1 (100)	1 (100) - Class I	3

**Table 4:** Summary of main surgical and clinical outcomes data

SURGICAL APPROACH *N* (%)	SETOA	20 (48.8)		
	SETOA + LO	20 (48.8)		
	ILTEA	1 (2.4)		
	TOT			42
OUTCOME	Surgical outcome	EoR	GTR	27 (73.0)
			STR	5 (13.5)
			PR	3 (8.1)
			Biopsy	2 (5.4)
			**TOT**	**37**
		CSF leak	1 (2.6)	
			**TOT**	**39**
		Permanent CNs deficit (III, IV, VI) and/or diplopia	0 (0)	
			**TOT**	**35**
		Other complications	Stroke	1 (2.6)
			Severe Bleeding	1 (2.6)
			Pseudomeningocele	1 (2.6)
			Postoperative hematoma	1 (2.6)
			**TOT**	**39**
	Clinical outcome	Postop VF	Preserved	23 (79.3)
			Stable	6 (20.7)
			Worsened	0 (0)
			**TOT**	**29**
		Seizure Control- Engel Outcome Scale	4 (100) – Class I	
			**TOT**	**4**
MEAN FU in months (range)	14.2 (2–35)			

Lastly, EoR outcomes were stratified by anatomical subregion (mbTR or pTL).

Among the 32 patients with mbTR lesions and available EoR data, 24 (75.0%) achieved GTR, 5 (15.6%) STR, and 3 (9.4%) PR. The reasons for incomplete resection, specified for 4 out of 8 patients in this group (50.0%), were as follows: multifocal lesions in two patients with AA (*n* = 2), excessive intraoperative bleeding in a patient with HPC (*n* = 1), and the proximity of a LGG to the basal ganglia, which the surgeons chose to preserve (*n* = 1). All of these procedures resulted in subtotal resection. However, in one of the two AA cases, the surgical plan did not include GTR.

In the pTL group, which included 5 patients, GTR was achieved in 3 cases (60.0%). In the remaining 2 patients, the surgical plan did not include GTR, as they underwent ETOA for biopsy (2, 40.0%).

Given that EoR is a meaningful parameter when the surgical goal is lesion resection, an additional analysis was performed excluding the two cases with planned biopsy, resulting in a cohort of 35 patients: 32 with mbTR involvement and 3 with lesions affecting the pTL.

GTR was achieved in 77.1% of cases (*n* = 27), STR in 14.3% (*n* = 5), and PR in 3 patients, representing 8.6% of the cohort. GTR was achieved in all three patients with pTL lesions (Table 5).

**Table 5:** Summary of surgical outcomes stratified by anatomical location

Location	EoR				Reasons for incomplete resection (*N*)
	GTR *N* (%)	STR *N* (%)	PR *N* (%)	Biopsy *N* (%)	
mbTR	24 (75.0)	5 (15.6)	3 (9.4)	0 (0)	Multifocal lesion (2) Excessive bleeding (1) Proximity to critical structures (1)
pTL	3 (60.0)	0 (0)	0 (0)	2 (40.0)	Planned biopsy (2)

The reconstruction technique was reported in 6 studies (46.1%) (Table 6). In 5 of these, the dural defect was repaired using a double-button technique with inlay and onlay patches. The materials used included autologous fascia lata, abdominal fat, and artificial dural substitutes. Chen et al. described a different reconstruction technique. The dura was loosely reapproximated using a 6− 0 absorbable suture. A free local muscle graft was then applied and sealed with an artificial dural substitute. Regarding additional perioperative procedures, Chen et al. preoperatively placed a frontal external ventricular drain, whereas Yoo et al. placed a lumbar drain postoperatively.

**Table 6:** Summarizes the reconstruction techniques used in the articles included in the systematic review

Author and year	Included Patients (*N*)	Reconstruction tecnique	Graft	Additional perioperative procedures
Chen et al., 2015 [27]	2	Dural defect suture and graft position	Free local muscle graft; sheet of smooth nylon.	Preoperative EVD
Jeon et al., 2018 [30]	1	Double-button technique (inlay/onlay patch)	Autologous fascia or acellular allogenic dermis; porous polyethylene implant	
Lee et al., 2019 [31]	1	Double-button technique (inlay/onlay patch)	Autologous fascia or acellular allogenic dermis	
Gerges et al., 2020 [23]	1	NA	NA	
Yoo et al., 2021 [22]	7	Double-button Technique (inlay/onlay patch)	Collagen matrix; abdominal fat; porous polyethylene implant	Postoperative LD
Park et al., 2021 [24]	7	NA	NA	
Lee et al., 2022 [32]	1	NA	NA	
Tanoue et al., 2024 [33]	1	Double-button technique (inlay/onlay patch)	Artificial dural substitute; Abdominal fat	
Carbone et al. 2024 [34]	1	NA	NA	
Iwami et al., 2024 [25]	2	NA	NA	
Torregrossa et al., 2025 [35]	1	NA	NA	
Jeon et al., 2025 [26]	15	Double-button technique (inlay/onlay patch)	Collagen matrix or acellular dermal matrix; Abdominal fat	
Maghalashvili et al., 2025 [36]	1	NA	NA	

EVD= external ventricular drain; LD= lumbar drain

AA=anaplastic astrocytoma; ACC = adenoid cystic carcinoma; AO=anaplastic oligodendroglioma; bFL= basal frontal lobe; CCM= cerebral cavernous malformation; DC=dermoid cyst; EoR= extent of resection; FU=follow-up; GBM= glioblastoma multiforme; GTR = gross total resection; HPC= hemangiopericytoma; ILTEA= inferolateral transorbital endoscopic approach; LGG = low grade glioma; LO = lateral orbitotomy; mbTR= mediobasal temporal region; MTX= metastasis; MVNT= multinodular and vacuolating neuronal tumor; NA = not available;; PR= partial resection; PC= pharyngeal carcinoma; PCN= plasma cell neoplasm; PXA = pleomorphic xanthoastrocytoma; pTL= temporal pole; RCC = renal cell carcinoma; SETOA= superior eyelid endoscopic transorbital approach; STR= subtotal resection; VF= visual field

Definitions

TOT: total number of patients for whom this specific item is available

GTR: Gross-total resection was defined as no evidence of residual tumor on the postoperative MRI

STR: Subtotal resection was defined as more than 90% of tumor removal on the postoperative MRI

PR: Partial resection was defined as less than 90% of tumor removal on the postoperative MRI

**Postop Visual field**

Postop VF was defined as “Preserved” if the preop VF was intact and there were no VF deficits after surgery Postop VF was defined as “Stable” if the preop VF was worsened (Jeon et al.,2025) or not available (Jeon et al., 2018; Gerges et al., 2020; Kim et al., 2021) but there were no new VF deficits after surgery

### Illustrative cases

#### Case 1 (mbTR Astrocytoma)

A 76-year-old man presented to the neurosurgery department complaining of confusion and amnesia for the past two years. Over the preceding three months, he had experienced several episodes of absence seizures. He had been followed by the neurology department, where limbic encephalitis was initially suspected. Follow-up MRI demonstrated an intra-axial lesion of the right hippocampus extending toward the crus of the right fornix, primarily suspected for mbTR glioma. DTI was performed to assess the relationship between the lesion and adjacent white matter tracts for preoperative planning.

Surgical resection with SETOA + LO was performed.

**Fig. 2:**
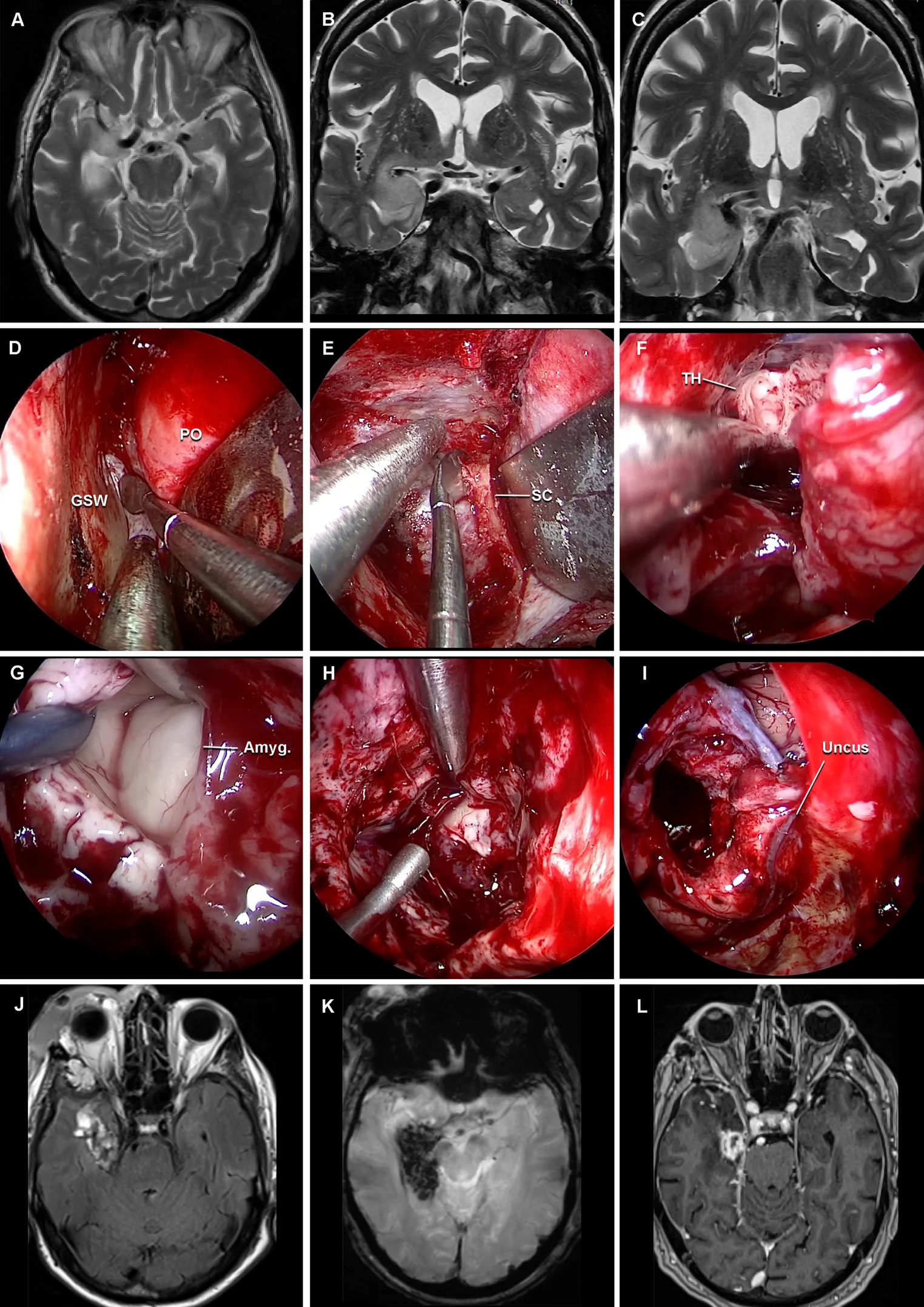
Illustrative case 1 Axial and coronal MRI scans showing an intra-axial, T2-hyperintense lesion in the right hippocampus extending toward the crus of the right fornix (A, B, C). Intraoperative view showing the lateral orbital wall subperiosteal dissection (D). The GSW is then drilled in a lateral-to-medial direction, followed by a LO. Afterward, the sagittal crest is removed (E). After dural opening, an inferior temporal corticectomy is performed, and intraparenchymal dissection is continued until the temporal horn of the lateral ventricle is identified (F). Tumor removal is started, and the amygdala is visualized (G). Tumor removal is carried on with an aspirator tip (H). Lastly, the uncus is exposed and partially removed (I). Axial postoperative FLAIR, SWI, and post-contrast T1-weighted MRI sequences (respectively) showing the surgical cavity; GTR of a WHO grade III astrocytoma is achieved (J, K, L). *Abbreviations*: Amyg.: Amygdala; GSW: Greater Sphenoid Wing; PO: Periorbita; SC: Sagittal Crest; TH: Temporal Horn of the lateral ventricle

GTR was achieved, and histopathological examination confirmed a World Health Organization (WHO) grade III astrocytoma. On postoperative day 7 the patient developed a mild wound fluid leakage and CSF leak was suspected; however, the revision craniotomy did not show any leak. The patient was discharged on postoperative day 30 and referred to the neuro-oncology team **(**Fig. 2, Video 1).

#### Case 2 (Multifocal Temporal Glioblastoma)

A 58-year-old woman presented with progressive behavioral and personality changes.

MRI demonstrated a highly contrast-enhancing multifocal lesion with extensive surrounding edema, involving the right temporal pole with posterior and superior extension into the ipsilateral temporal lobe.

Surgical resection with SETOA + LO was planned.

GTR was achieved, and histopathological examination confirmed a World Health Organization (WHO) grade IV glioma.

**Fig. 3:**
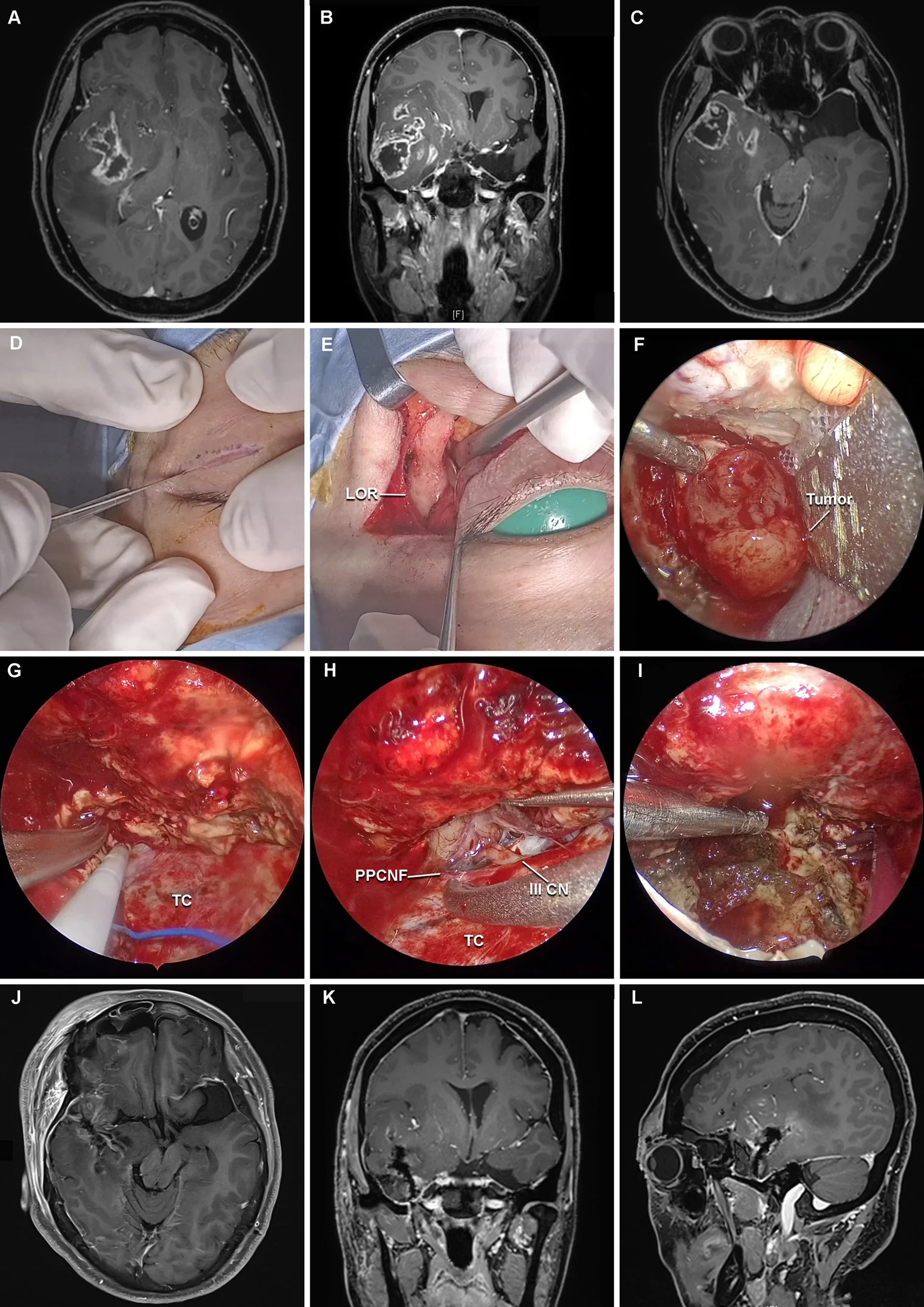
Illustrative case 2. Axial and coronal T1-weighted MRI scans showing a large, multifocal, contrast-enhancing mass involving the right temporal pole with posterior and superior extension into the ipsilateral temporal lobe (**A**, **B**, **C**). Intraoperative view showing a superior eyelid crease incision (**D**). A subperiosteal dissection toward the margins of the superior and inferior orbital fissures is then performed in a superolateral-to-posteromedial direction; note the use of a corneal protector (**E**). The periorbita is protected, and the globe is retracted using a self-retaining malleable retractor. After a wide dural opening, the lesion is visualized (**F**). Under neuronavigation guidance, the tumor is resected using an ultrasonic aspirator (**G**). After tumor removal, the oculomotor–tentorial triangle is exposed (**H**). Hemostasis is performed with bipolar forceps (**I**). Axial, coronal, and sagittal postoperative T1-weighted MRI scans demonstrate GTR of the tumor (**J**, **K**, **L**). *Abbreviations*: III CN: Oculomotor nerve (Third cranial nerve); LOR: Lateral Orbital Rim; TC: Tentorium Cerebelli; PPCNF: Posterior Petroclinoid Fold

The patient was discharged on postoperative day 3 and subsequently underwent concurrent chemoradiotherapy with temozolomide (Fig. 3, Video 1).

## Discussion

This systematic review synthesizes current evidence on the application of ETOAs for the surgical treatment of intraparenchymal temporal lesions. Analysis of 13 eligible studies encompassing 41 patients reveals that ETOA, originally devised for skull base pathologies, has been successfully adapted for selected intraparenchymal temporal lesions. It should be noted that a single case reporting an insular glioma treated with an ETOA was identified; however, due to the limited evidence from a single-patient report, it was not included in this review [28]. Most lesions were in the mbTR, with GTR achieved in approximately 73% of cases and no postoperative VF deterioration observed. The superior eyelid transorbital approach (SETOA) and the combined superior eyelid approach with lateral orbitotomy (SETOA + LO) represented the predominant surgical techniques utilized [8]. The orbit’s unique anatomical proximity to both the anterior and middle cranial fossae renders it an ideal conduit for reaching deep-seated temporal lesions [10]. Its anteromedial position relative to the temporal lobe enables a direct trajectory toward the temporal pole and mediobasal temporal structures [17, 24, 27, 44, 45]. The superior eyelid crease incision, used in nearly all the reported cases, provides a cosmetically discreet and minimally invasive entry point, enabling access to these regions without necessitating wide craniotomies or cortical transgression. This is particularly beneficial when targeting anteriorly located lesions such as those in the temporal pole or basal frontal lobe [33, 36, 41–43]. Of note, three studies reporting patients with intraparenchymal basal frontal lobe lesions treated using the ETOA were identified in the literature; however, they were excluded, as they were not pertinent to the anatomical region examined in this study [41–43].

One of the most significant advantages of ETOAs in this setting is the preservation of the lateral neocortex. Traditional MTAs often require disruption of lateral temporal gyri, venous structures, and subcortical white matter pathways. Such manipulation can lead to postoperative cognitive and visual deficits, particularly when the optic radiations are involved [46, 47]. In contrast, the anterior-to-posterior trajectory of the ETOAs follows the natural long axis of the temporo-insular regions, allowing for selective resection while sparing lateral cortex [24, 26, 28]. While the SETOA provides initial access to the anterior and medial temporal lobe, the SETOA + LO significantly enhances the working angle and instrument maneuverability particularly when approaching posterior or superiorly located lesions [8, 48]. This modification was employed in half of the reviewed cases, often in those targeting more medially or posteriorly situated tumors. The angle and depth of the surgical corridor are key considerations in planning an ETOA [7, 26, 48].

However, broader functional outcomes, including cognition, are not systematically addressed in the studies. While no postoperative cognitive deficits are reported, formal pre- and postoperative neuropsychological assessments were not performed, leaving ETOA’s validity in achieving broader functional preservation unproven in clinical practice.

Preservation of visual function is a paramount concern in temporal lobe surgery, especially considering the close anatomical relationship between the mbTR and the optic radiations, particularly Meyer’s loop [26, 46, 47]. Visual field deficits are reported in up to 76% of patients undergoing anterior temporal lobectomy via MTAs [46, 49–51]. The ETOAs, however, minimize the risk of such deficits by limiting neocortical retraction and enabling entry into the medial temporal lobe from a more anterior and inferior angle [17, 24, 26–28]. When paired with preoperative DTI tractography, this corridor allows precise mapping of the optic radiations and Meyer’s loop, guiding surgical trajectories to avoid these critical fibers [26, 52, 53]. In accordance, Jeon et al. adopted DTI as part of their surgical planning, achieving optimal postoperative VF outcomes [26].

In addition to DTI, high-definition endoscopy provides enhanced illumination and magnification within narrow working corridors, facilitating subpial dissection and identification of microanatomical structures. This is especially valuable in navigating the densely vascularized mbTR, which is bordered medially by critical neurovascular structures including the anterior choroidal artery, posterior cerebral artery, and the basal vein of Rosenthal [10, 17, 24, 26]. Successful ETOA surgeries in this region depend on a thorough understanding of three-dimensional microsurgical anatomy and a meticulous subpial technique. Several of the reviewed studies employed neuronavigation as standard adjunct, which may have contributed to the postoperative visual field outcome observed in this review [22, 25–27, 30, 31, 34, 36]. Notably, DTI and neuronavigation are tools commonly employed in MTAs as well; therefore, the benefits of these technologies are available in both of the approaches discussed.

Considering the patient cohort with available postoperative VF data, as previously reported in the Results section, only half of the patients underwent postoperative VF evaluation using both clinical ophthalmological examination and OR tractography documenting intact optic radiations, while only two patients were evaluated solely through clinical VF examination. For the remaining 13 patients, the VF assessment method was not available. Despite this encouraging rate of VF preservation, we acknowledge a lack of consistency in the VF outcome data.

An additional advantage of the ETOA, particularly relevant in the context of temporomesial lesions, is the avoidance of temporalis muscle dissection. When considering that conventional transcranial approaches to temporal lesions require temporalis muscle transgression—associated in the literature with postoperative headache, pain and dysfunction of the masticatory muscles, limited mouth opening, sensory disturbances in the temporal region, and even weather sensitivity—the possibility of the transorbital route to entirely preserve temporalis muscle integrity represents a meaningful functional and aesthetic benefit [7, 54].

Despite these advantages, ETOA is not devoid of limitations. The constrained working corridor imposes ergonomic challenges, especially during prolonged resections or when dealing with firm or high vascularized lesions. The steep learning curve, coupled with the need for high anatomical precision, underscores the importance of surgeon experience and careful case selection. Furthermore, CSF leakage, though infrequent (2.6%), remains a potential complication, particularly when dura or skull base integrity is compromised. Meticulous reconstruction - typically involving multilayer closure with fat grafts, collagen matrices, and fibrin sealants - is crucial to preventing postoperative CSF leaks and cosmetic deformities [29, 55, 56]. It should also be noted that, in many cases, an additional incision is required to harvest an abdominal fat graft [57].

In their case series, Yoo et al. reported a postoperative CSF leak. The reconstruction was performed with inlay/onlay abdominal fat graft sealed with fibrin glue. The CSF leak was managed with lumbar drainage, and the patient recovered within one week without neurological complications [22].

Importantly, the long-term oncological efficacy of ETOAs for gliomas remains to be fully elucidated, given the limited follow-up durations in available studies (mean 14.2 months). While early results demonstrate acceptable GTR rates and seizure control in cases with mbTR involvement, more extensive data are required to confirm its comparability with conventional approaches in terms of survival and recurrence [24, 26, 27].

In a recent case series of intra-axial tumors involving the mbTR treated via MTAs, the reported GTR rate was 74.5%, with a mortality rate of 1.3% and a surgical complication rate of 7.1%.

However, the patients included in the cited study are not directly comparable to the cohort analyzed in this review. First, most lesions were gliomas, whereas the pathologies present in this review are more heterogeneous. Second, lesions with insular involvement were included, whereas no insular lesions were present in our study [58].

Furthermore, there are also differences in the outcome reporting methods. Therefore, caution should be exercised when comparing these two studies, particularly in light of differences in patient selection and outcome reporting methodology.

Other older studies in the literature focused on non-lesional epilepsy and are likewise not directly comparable to the cohort analyzed in this review [46, 49–51].

Another important consideration is that ETOA precludes awake mapping. However, the surgical trajectory along the temporo-insular axis allows preservation of critical temporal white matter tracts, including language-related pathways. In their case series, Alkassm et al. performed awake craniotomy in only 2 of 157 patients in the cohort [58].

Nevertheless, the incompatibility of ETOA with awake mapping represents a limitation, and an MTA may be preferred in cases in which intraoperative mapping is required to achieve maximal safe resection.

Finally, current visualization platforms present differential integration of intraoperative fluorescence. Both the surgical microscope and exoscope for transcranial approaches now routinely support the use of fluorescein sodium, indocyanine green (ICG), and 5-aminolevulinic acid (5-ALA), facilitating tumor delineation and vascular assessment [59–62]. In contrast, while most endoscopic systems reliably support fluorescein and ICG, uniform compatibility with 5-ALA has historically been limited [60, 63]. However, recent evidence demonstrates that endoscopic visualization of 5-ALA–induced protoporphyrin IX fluorescence is becoming a concrete and increasingly adopted reality, with promising results reported in high-grade glioma surgery [64, 65].

### Limitations of the study

The overall number of included studies and patients remains limited, reflecting the early stage of clinical experience with ETOA for intraparenchymal temporal lesions. Most studies were retrospective case series with small sample sizes or case reports, introducing potential selection bias and limiting the generalizability of findings. The available data did not allow for a systematic analysis of tumor size in relation to surgical outcome. Tumor diameter or volumetric assessment is a critical determinant of surgical feasibility, extent of resection, and complication profile. Smaller temporopolar lesions, such as cavernous malformations, are inherently more amenable to gross-total resection, whereas larger gliomas with deep extension may pose significant challenges. The absence of size-based stratification therefore limits the interpretation of the reported resection rates. Furthermore, the reasons for incomplete resection were seldom specified in the included studies. In clinical practice, residual tumor often reflects proximity to critical structures such as the internal capsule, corticospinal tracts, or deep vascular networks, rather than purely technical limitations of the approach. A more detailed understanding of these limiting factors would provide valuable guidance for surgical planning and case selection. Additionally, there was substantial heterogeneity in lesion types, anatomical locations and outcome reporting, which precluded a robust quantitative synthesis or meta-analysis.

The lack of standardized outcome measures, particularly in assessing visual field deficits, seizure control and other functional outcomes, further complicates cross-study comparisons and thus the implications of this review. More specifically, the postoperative VF assessment methodology is missing for more than 40% of the patients, and details regarding the exact ophthalmological examination performed, whether bedside examination or perimetry, were not reported.

Therefore, the ability of ETOA to spare the optic radiations has not yet been definitively demonstrated in clinical practice, despite encouraging preliminary results.

Regarding functional outcomes beyond visual field preservation, a further limitation of this study is the absence of formal pre- and postoperative neuropsychological assessments, as previously noted.

Lastly, follow-up durations were relatively short in many studies, limiting long-term assessment of recurrence rates and neurological function.

Considering the findings of this review, the authors suggest that future clinical studies on ETOA for temporal lobe lesions include standardized pre- and postoperative neuropsychological assessments, as well as visual field perimetry and optic radiation tractography.

## Conclusions

Endoscopic transorbital approaches are rapidly evolving from a skull base technique to a versatile route for intraparenchymal lesions. Its anterior-to-posterior trajectory along the temporo-insular axis, combined with the possibility of spare eloquent cortical/subcortical pathways and its synergy with advanced neuroimaging techniques, make it a viable option in selected cases. As surgery increasingly shifts toward function-preserving strategies, ETOA embodies the spirit of exploration expressed in the motto “Navigare necesse est” - the exhortation which, according to Plutarch, persuaded sailors to continue rowing toward Rome despite adverse weather - symbolizing the pursuit of new, innovative pathways through anatomically complex regions.

## Supplementary Information

Below is the link to the electronic supplementary material.

Supplementary Material 1 (DOCX 196 KB)

Supplementary Material 2 (DOCX 56.0 KB)

Video 1: Operative video showing the two surgical cases

Supplementary Material 3 (mp4 517 MB)

## Data Availability

This systematic review was conducted in accordance with the Preferred Reporting Items for Systematic Reviews and Meta-Analyses (PRISMA) guidelines. Two reviewers (E.A. and L.G.) independently performed a comprehensive search of the PubMed, Scopus and Ovid MEDLINE databases. The initial search was conducted on March 2, 2025, with a final update on April 18, 2025.The search strategy utilized combinations of keywords and MeSH terms, including “endoscopic transorbital approach,” “ETOA,” “temporal lobe,” “intraparenchymal lesions,” “mediobasal temporal region,” “temporal pole,” “insular cortex,” “tumor resection,” and “neurosurgery.” Boolean operators (AND/OR) were used to structure the query as follows: (“endoscopic transorbital approach” OR “ETOA”) AND (“temporal lobe” OR “mediobasal temporal” OR “temporal pole” OR “insula”) AND (“tumor” OR “resection” OR “surgery”) (Supplementary Material 1). Additional relevant studies were identified through manual screening of reference lists from selected articles.
