# Supplementary material for: Navigare necesse est: The transorbital route to intraparenchymal temporal lesions
Source: Neurosurg Rev. 2026 Aug 25;49(1):538. doi: 10.1007/s10143-026-04402-x (PMC13506574; doi:10.1007/s10143-026-04402-x)
Supplement: Supplementary file 1 [file 10143_2026_4402_MOESM1_ESM.docx]

**Supplementary material 1**

PUBMED=129 (1 report included via Cross-reference)

("transorbital") AND (("Temporal Lobe"[Mesh]) OR ("mesial temporal") OR ("medial temporal") OR ("mediobasal temporal") OR ("MTL") OR ("MTR") OR ("Limbic System"[Mesh]) OR ("insula") OR ("Central Nervous System Neoplasms"[Mesh]) OR ("glioma") OR ("glioblastoma") OR ("GBM") OR ("Neoplasm Metastasis"[Mesh]) OR ("Epilepsy, Temporal Lobe"[Mesh]) OR ("cavernoma") OR ("Hemangioma, Cavernous"[Mesh]) OR ("Central Nervous System Vascular Malformations"[Mesh]))

SCOPUS n=334	(370-36) 36 excluded by automation tools

("endoscopic transorbital" OR "transorbital approach") AND ("temporal lobe" OR "temporal region" OR "mesial temporal " OR "mediobasal temporal" OR "MTR" OR "MTL" OR "insula" OR "amygdala" OR "hippocampus" OR "entorhinal cortex" OR "parahippocampal gyrus" OR "intraparenchymal" OR "intra-axial" OR "glioma" OR "glioblastoma" OR "GBM" OR "metastasis" OR "epilepsy" OR "cavernous malformations" OR "cavernoma" OR "cavernous hemangioma") AND ( LIMIT-TO ( DOCTYPE,"ar" ) OR LIMIT-TO ( DOCTYPE,"re" ) )

OVID medline n=159 (228-69) 69 excluded by automation tools

(("endoscopic transorbital" or "transorbital approach") and ("temporal lobe" or "temporal region" or "mesial temporal " or "mediobasal temporal" or "MTR" or "MTL" or "insula" or "amygdala" or "hippocampus" or "entorhinal cortex" or "parahippocampal gyrus" or "intraparenchymal" or "intra-axial" or "glioma" or "glioblastoma" or "GBM" or "metastasis" or "epilepsy" or "cavernous malformations" or "cavernoma" or "cavernous hemangioma")).mp. [mp=tx, bt, ti, ab, ct, sh, ot, nm, hw, fx, kf, ox, px, rx, an, ui, ds, on, sy, ux, mx]

**Supplementary material 2**

**Supplementary material 3**

| Study | Year | Selection (4) | Comparability (2) | Outcome (3) | Total Score | Quality |
| --- | --- | --- | --- | --- | --- | --- |
| Chen et al. | 2015 | 3 | 2 | 3 | 8 | High |
| Jeon et al. | 2018 | 4 | 2 | 3 | 9 | High |
| Lee et al. | 2019 | 3 | 2 | 2 | 7 | High |
| Gerges et al. | 2020 | 3 | 2 | 3 | 8 | High |
| Yoo et al. | 2021 | 4 | 2 | 3 | 9 | High |
| Park et al. | 2021 | 3 | 1 | 3 | 7 | High |
| Lee et al. | 2022 | 4 | 1 | 3 | 8 | High |
| Tanoue et al. | 2024 | 4 | 2 | 3 | 9 | High |
| Carbone et al. | 2024 | 3 | 2 | 3 | 8 | High |
| Iwami et al. | 2024 | 3 | 1 | 3 | 7 | High |
| Torregrossa et al. | 2025 | 3 | 2 | 3 | 8 | High |
| Jeon et al. | 2025 | 4 | 2 | 3 | 9 | High |
| Maghalashvili et al. | 2025 | 3 | 2 | 2 | 7 | High |
