# Supplementary material for: Navigare necesse est: The transorbital route to intraparenchymal temporal lesions
Source: Neurosurg Rev. 2026 Aug 25;49(1):538. doi: 10.1007/s10143-026-04402-x (PMC13506574; doi:10.1007/s10143-026-04402-x)
Supplement: Supplementary file 2 [file 10143_2026_4402_MOESM2_ESM.docx]

**Identification of studies via databases and registers**

Records removed *before screening*:

Duplicate records removed (n =109)

Records marked as ineligible by automation tools (n = 105)

Records removed for other reasons (n = 0)

Records identified from:

PubMed (n = 129)

Ovid MEDLINE (n = 228)

Scopus (n = 370)

**Identification**

Records screened

(n = 513)

Records excluded

(n = 491)

Reports sought for retrieval

(n = 22)

Reports not retrieved

(n = 0)

**Screening**

Reports excluded:

Not relevant to the research topic (n = 4)

Missing methods, results or details (n =0)

Overview, systematic literature review and meta-analysis (n = 5)

Reports assessed for eligibility

(n = 22)

Studies included in review

(n = 13)

Reports of included studies

(n = 13)

**Included**

Copyright © Agosti et al. 2025

*Consider, if feasible to do so, reporting the number of records identified from each database or register searched (rather than the total number across all databases/registers).

**If automation tools were used, indicate how many records were excluded by a human and how many were excluded by automation tools.

*From:* Page MJ, McKenzie JE, Bossuyt PM, Boutron I, Hoffmann TC, Mulrow CD, et al. The PRISMA 2020 statement: an updated guideline for reporting systematic reviews. BMJ 2021;372:n71. doi: 10.1136/bmj.n71

For more information, visit: <http://www.prisma-statement.org/>
